# Immunogenicity of a new enhanced tetanus-reduced dose diphtheria-acellular pertussis (Tdap) vaccine against *Bordetella pertussis* in a murine model

**DOI:** 10.1186/s12865-021-00457-1

**Published:** 2021-10-12

**Authors:** Kyu Ri Kang, Dong Ho Huh, Ji Ahn Kim, Jin Han Kang

**Affiliations:** 1 The Vaccine Bio Research Institute, College of Medicine, The Catholic University of Korea, Seoul, Annex to Seoul Saint Mary Hospital, 222 Banpo-daero, Seocho-gu, Seoul, 06591 Korea; 2 Department of Pediatrics, Seoul St. Mary’s Hospital, College of Medicine, The Catholic University of Korea, 222 Banpo-daero, Seocho-gu, Seoul, 06591 Korea

**Keywords:** Tetanus-reduced dose diphtheria-acellular pertussis vaccine, Immunogenicity, Mouse study

## Abstract

**Background:**

The necessity of the tetanus-reduced dose diphtheria-acellular pertussis (Tdap) vaccine in adolescence and adults has been emphasized since the resurgence of small-scale pertussis in Korea and worldwide due to the waning effect of the vaccine and variant pathogenic stains in the late 1990s. GreenCross Pharma (GC Pharma), a Korean company, developed the Tdap vaccine GC3111 in 2010. Recently, they enhanced the vaccine, GC3111, produced previously in 2010 to reinforce the antibody response against filamentous hemagglutinin (FHA). In this study, immunogenicity and efficacy of the enhanced Tdap vaccine compared and evaluated with two Tdap vaccines, GC3111 vaccine produced in 2010 previously and commercially available Tdap vaccine in a murine model.

**Methods:**

Two tests groups and positive control group of Balb/c mice were primed with two doses of the diphtheria-tetanus-acellular pertussis (DTaP) vaccine followed by a single booster Tdap vaccine at 9 week using the commercially available Tdap vaccine or 2 Tdap vaccines from GC Pharma (GC3111, enhanced GC3111). Humoral response was assessed 1 week before and 2 and 4 weeks after Tdap booster vaccination. The enhanced GC3111 generated similar humoral response compare to the commercial vaccine for filamentous hemagglutinin (FHA). The interferon gamma (IFN-γ) (Th1), interleukin 5 (IL-5) (Th2) and interleukin 17 (IL-17) (Th17) cytokines were assessed 4 weeks after booster vaccination by stimulation with three simulators: heat inactivated *Bordetella pertussis* (hBp), vaccine antigens, and hBp mixed with antigens (hBp + antigen). A bacterial challenge test was performed 4 weeks after booster vaccination.

**Results:**

Regarding cell-mediated immunity, cytokine secretion differed among the three simulators. However, no difference was found between two test groups and positive control group. All the vaccinated groups indicated a Th1 or Th1/Th2 response. On Day 5 post-bacterial challenge, *B. pertussis* colonies were absent in the lungs in two test groups and positive control group.

**Conclusions:**

Our results confirmed the immunogenicity of GC Pharma’s Tdap vaccine; enhanced GC3111 was equivalent to the presently used commercial vaccine in terms of humoral response as well as cell-mediated cytokine expression.

**Supplementary Information:**

The online version contains supplementary material available at 10.1186/s12865-021-00457-1.

## Background

Sporadic outbreaks of pertussis among adolescents and adults have continuously been reported worldwide, including in advanced countries such as Europe, Australia, the USA, and Japan, where the rate of vaccination is above 90%, and yet, the disease is spreading steadily [1–3]. The reasons for the increased occurrence of pertussis include the following. The antibodies produced after acellular pertussis vaccination last for approximately 5–6 years, [4, 5], and thus, the likelihood of reinfection increases during adolescence and adulthood because of the waning effect of acellular pertussis vaccine. Moreover, the genetic changes in circulating strains of *Bordetella pertussis* (*B. pertussis*) such as pertactin (PRN)-deficient variants [6–8] or pertussis toxin (PT) promoter alleles [9] in advanced countries, may have caused the reemergence of pertussis. In addition, the pertussis vaccination rate reported to be low in adolescents and adults. Furthermore, resistance to macrolide antibiotics following outbreaks is a major challenge in some countries [10]. Therefore, to address the epidemiological changes, the tetanus-reduced dose diphtheria-acellular pertussis (Tdap) vaccination should be encouraged to adolescents and adults, and simultaneously, new vaccines that protect against variant strains should be developed.

In Korea, the Korea National Institution of Health established the laboratory diagnostics of pertussis in 1999, and since then, only 18 incidences observed annually until 2008. However, the numbers increased subsequently, with 66 cases in 2009, 27 in 2010, and 97 cases observed in 2011 [11]. In the first half of 2012, sudden small outbreaks reported around the schools in certain regions. Since then, small sporadic outbreaks have continued to occur with a steady increasing trend. With epidemiological changes in Korea, immunization of adolescents and adults with Tdap vaccine is necessary [12, 13]. Currently, no Tdap vaccine manufacturer exists in Korea, so the country relies on imported vaccines. Therefore, vaccination is limited, as the vaccine is not easily available. To resolve this issue, Green Cross Pharma (GC Pharma, Yongin, Korea) began developing a Tdap vaccine (GC3111) in 2010 and began Phase I and IIa clinical trials in 2017. During the trials, the antibody titre against PT, filamentous hemagglutinin antigen (FHA), and PRN antigens revealed positive seroconversion and seroprotection after vaccination; however, the vaccine induced a lower titre level of the antibody to FHA compared to the commercially available control vaccine Boostrix™ (GlaxoSmithKlein, Rixensart, Belgium) [14]. Based on this finding, an enhanced GC3111 Tdap vaccine with increased antigen volume was developed by improving FHA inactivation and purification. The present study aimed to investigate whether the enhanced vaccine (enhanced GC3111) had improved immunological outcomes and efficacy by comparing the vaccine produced in 2010 (GC3111) and the existing commercial vaccine using an animal-based model prior to conducting human trials.

## Methods

### Mice

During the animal research period, the mouse were housed in filter-top cages under semi-specific pathogen free conditions and food and water are available freely. All animal research procedures performed in accordance with the Laboratory Animals Welfare Act, the Guide for the Care and Use of Laboratory Animals and the Guidelines and Policies for Rodent Experiments provided by the IACUC (Institutional Animal Care and Use Committee) in the School of Medicine, The Catholic University of Korea. (Approval number: CUMS-2019-0100-01).

### Vaccination

**Fig. 1:**
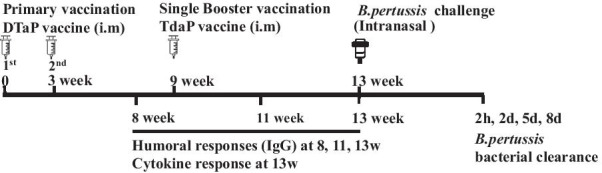
Schemes for vaccination, challenge and assay of study (DTaP = diphtheria-tetanus-acellular pertussis, Tdap = tetanus-reduced dose diphtheria-acellular pertussis)

The study conducted according to previous murine model studies at our laboratory at the Vaccine Bio Research Institute [15, 16]. Four-week-old BALB/c female mice from Orient-bio Co., Ltd. (Seongnam, Korea) used in the study. All mice were vaccinated with two doses of primary diphtheria-tetanus-acellular pertussis (DTaP) vaccine at 3-week intervals with 0.125 mL (one-fourth of the human dose) via intramuscular (quadriceps muscle) injection and vaccinated except for the negative control group, which was injected with saline before booster vaccination. DTaP vaccines were provided by GC Pharma and composed of 25 Lf diphtheria toxoid (DT), 10 Lf tetanus toxoid (TT), 25 μg PT, 25 μg FHA 25 μg and 8 μg PRN per 0.5 mL. The mice divided into 4 groups (30 mice per group) according to booster vaccine types: negative control injected with saline, positive control with licensed Tdap vaccine (Boostrix™) and two study groups; one study group with the Tdap vaccine GC3111 produced in 2010 by GC Pharma and the other study group with the enhanced FHA antigen of GC 3111 vaccine (Table 1). All Tdap vaccine components were equivalent to DT 2 IU, TT 20 IU, PT 8 μg, FHA 8 μg, and PRN 2.5 μg per 0.5 mL and vaccinated with 0.125 mL via intramuscular injection. The vaccination and assay schedule described in Fig. 1.

**Table 1:** Characteristics of study groups

Group	Primary (1st, 2nd) vaccine	Booster vaccine
Negative control	Saline	Saline
Positive control	DTaP from GC pharma	Commercially available Tdap
GC3111	DTaP from GC pharma	GC3111 Tdap produced in 2010
Enhanced GC3111	DTaP from GC pharma	GC3111 Tdap FHA enhanced

DTaP = diphtheria-tetanus-acellular pertussis, GC Pharma = Green Cross Pharma, Tdap = tetanus-reduced dose diphtheria-acellular pertussis, FHA = filamentous hemagglutinin

### Humoral immune response assay

Blood samples from the retro-bulbar venous plexuses were collected in each group at 1 week before booster vaccination (n = 6 per group) and 2, 4 week after booster vaccination (n = 10). When sampling the blood, all mice were anesthetized with tiletamine, zolazepam and xylazine via intra peritoneal injection except last sampling. At 4 week after booster vaccination, mice were euthanized by 2% isoflurane inhalation while sampling and sacrificed via CO_2_ inhalation. The humoral immunogenicity against pertussis antigens (anti-PT IgG, anti-FHA IgG and anti-PRN IgG) was evaluated by commercially available ELISA kits (Alpha Diagnostic International Inc., San Antonio, TX, USA). Additionally, anti-diphtheria toxoid (DT) IgG and anti-tetanus toxoid (TT) IgG titres were measured using commercially available ELISA kits (Alpha Diagnostic International Inc. San Antonio, TX, USA). Results were analysed through optimal density using an Epoch ELISA plate reader (BioTek Instrumetns Inc., Winooski, VT, USA). Antibody titres of each tested antigen compared between groups at each time point.

### Cell-mediated immune response assay

Four weeks after the booster vaccination, mouse spleen cells (n = 5 per group) were resuspended in RPMI-1640 (HyClone, GE Healthcare Life Sciences, SouthLogan, Utah, USA) medium containing penicillin, streptomycin, and 10% FBS. For cell-based experiments, 1 μg/mL pokeweed mitogen (PWM; Sigma-Aldrich, St. Louis, MO, USA) was used as a positive control, and the following three stimulators were tested: 1 × 10^6^ colony forming units (CFUs) /mL of heat inactivated *B. pertussis* (hBp), PT (8 μg/mL), FHA (8 μg/mL) and PRN (4 μg/mL) vaccine antigens, and the mixture of the two (hBp + antigens). Splenocytes (5 × 10^6^ cells/mL) of each mice were added to 6-well plates (2 mL/well) and treated with three simulators separately and cultured for 3 days. Subsequently, the cytokine response was assessed by analysing the supernatant using ELISA kits (R&D Systems, Minneapolis, MN, USA).

### Bacterial challenge test

The protective efficacy against *B. pertussis* infection was assessed with intranasal clearance tests according to previous study [15–17]. The challenge *B. pertussis* strain obtained from a Korean adult pertussis patient was supplied from the Korean Centers for Disease Control & Prevention (KCDC) (No. 13674) and was inoculated at 4 weeks after booster vaccination. 6 × 10^6^ CFUs of *B. pertussis* suspended in 50μL of phosphate buffered saline (PBS) and injected intranasally. Four mice in each group at each time point were euthanized by 2% isoflurane inhalation and their lungs were extracted 2 h, 2 days, 5 days and 8 days after infection. The extracted whole lungs (5 lobes) were grinded with 10 mL of PBS and diluted to tenfold dilutions. Each diluted homogenate was cultured on Bordet-Gengou agar supplemented with 15% defibrinated horse blood and incubated for 5 days at 37 °C. CFUs on each media were determined and mean CFUs were compared between groups at each time point.

### Statistical analysis

All results are expressed as the means ± standard errors of the means (SEM) and compared by two-way ANOVA with Tukey’s multiple comparison test. Statistical analysis was performed using GraphPad Prism™ software v7.02 (GraphPad, San Diego, CA, USA), and statistical significance was defined as a *p* value (^*^p < 0.05, ^**^p < 0.01, ^***^p < 0.001, ^****^p < 0.0001).

## Results

### Humoral response

**Fig. 2:**
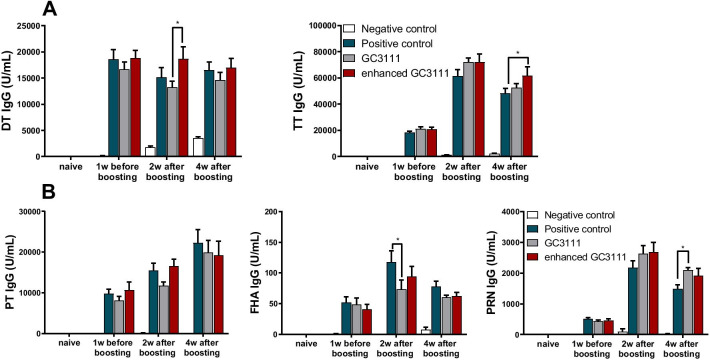
Humoral immune response. Humoral response was assessed 1 week before and 2 and 4 weeks after the booster vaccination. The results from commercially available ELISA kits are presented as the mean ± SEM (U/mL) in the graphs. The experiment was performed using five mice per group in the naïve groups and six mice per group 1 week before vaccination. In all other conditions, experiments were performed on 10 mice per group. Statistical differences were tested with two-way ANOVA and Tukey’s multiple comparison test. **A** IgG responses to diphtheria and tetanus were assessed by using commercially available ELISA kits. **B** Anti-PT, anti-FHA, and anti-PRN IgG titer levels were assessed by using commercially available kits. (SEM = standard errors of the means, PT = pertussis toxin, FHA = filamentous haemagglutinin, PRN = pertactin) (*p < 0.05, **p < 0.01, ***p < 0.001, ****p < 0.0001)

The humoral immune response was examined 1 week before (n = 6 per group) and 2 and 4 weeks (n = 10 per group) after the booster vaccination. In the anti-DT IgG, anti-TT IgG and anti-pertussis IgG humoral responses, the enhanced GC3111 group had equivalent titres compared to the positive control (*p* > 0.01, Fig. 2). Titre of two study groups and positive control group had highest titre at 4 weeks after boosting against for PT antigen but for FHA and PRN antigen had highest titre at 2 weeks after boosting (Fig. 2B). The mean titre of anti-PT IgG peaked to 22,270.00 U/mL in positive control group and 19,203.90 U/mL in enhanced GC3111 group at 4 weeks after boosting (Table 2). The mean titre against for FHA antigen had increased 2 weeks after boosting, 117.70 U/mL in positive control, 93.90 U/mL in enhanced GC3111 and 73.10 U/mL in GC3111 group (Table 2). In a similar way to FHA, anti-PRN IgG peaked 2 weeks after boosting, 2,181.00 U/mL in positive control and 2,686.10 U/mL in enhanced GC3111 group (Table 2).

**Table 2:** Humoral responses against pertussis toxin (PT), filamentous hemagglutinin (FHA), and pertactin (PRN) (mean ± SEM)

	Negative control	Positive control	GC3111	Enhanced GC3111
Anti-PT IgG (U/mL)				
Naïve (N = 5)	0.86 ± 0.17	0.55 ± 0.16	0.66 ± 0.16	0.82 ± 0.31
1w before booster vaccination (n = 6)	0	9,804.25 ± 1,061.82	8,102.17 ± 1,002.83	10,608.92 ± 2,022.17
2w after booster vaccination (N = 10)	92.20 ± 63.98	15,457.80 ± 1,781.44	11,734.00 ± 886.59	16,580.25 ± 1,663.38
4w after booster vaccination (N = 10)	0	22,270.00 ± 3,286.30	19,936.00 ± 2,972.66	19,203.90 ± 3,494.35
Anti-FHA IgG (U/mL)				
Naïve (N = 5)	0.01	0	0	0.02 ± 0.01
1w before booster vaccination (N = 6)	0.88 ± 0.22	51.41 ± 9.98	48.39 ± 11.00	40.74 ± 7.85
2w after booster vaccination (N = 10)	0	117.70 ± 17.95	73.10 ± 15.04	93.90 ± 16.80
4w after booster vaccination (N = 10)	7.50 ± 3.84	78.00 ± 8.79	60.50 ± 3.45	62.00 ± 6.35
Anti-PRN IgG (U/mL)				
Naïve (N = 5)	0	0	0	0
1w before booster vaccination (N = 6)	2.88 ± 2.80	510.42 ± 42.25	436.08 ± 38.92	456.75 ± 52.19
2w after booster vaccination (N = 10)	96.40 ± 96.40	2,181.00 ± 225.35	2,629.10 ± 272.59	2,686.10 ± 320.84
4w after booster vaccination (N = 10)	18.90 ± 18.90	1,491.50 ± 127.52	2,101.40 ± 73.77	1,918.10 ± 237.37

SEM = standard errors of the means

### Cell-mediated immune response

**Fig. 3:**
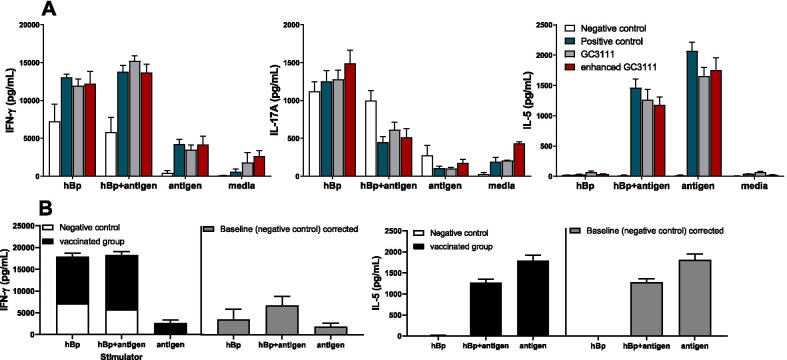
Cytokine response in each stimulator. Two and four weeks after booster vaccination, stimulation was carried out by three stimulators with heat-killed *B. pertussis* (hBp), PT, FHA, and PRN antigens or the mixture of the two (hBp + antigen) for 3 days (*n* = 5). The culture medium was used as a negative control, and 1 µg/mL PWM was used as a positive control. The results were obtained via commercially available cytokine ELISA kits and presented as the mean ± SEM (pg/mL). All results were baseline (medium only) corrected. Statistical differences were tested with 2-way ANOVA and Tukey’s multiple comparison test. **A** IFN-γ, IL-17A, and IL-5 cytokine levels were assessed for each stimulator. There were no statistically significant differences between the three vaccinated groups; however, different cytokines revealed various response levels according to the stimulator type. **B** The mean cytokine secretions of the three vaccinated groups, positive control group and two study groups had different levels by what stimulators used. When the mean cytokine levels were subtracted by the cytokine levels of the negative control group (saline injected) to correct baseline, Th1 (IFN-γ) immunity was confirmed by stimulator hBp. Th1 (IFN-γ) / Th2 (IL-5) polarization was confirmed by stimulator hBp + antigen and antigen respectively. (SEM = standard errors of the means, PT = pertussis toxin, FHA = filamentous haemagglutinin, PRN = pertactin) (*p < 0.05, **p < 0.01, ***p < 0.001, ****p < 0.0001)

Cell-mediated immunity (CMI) was evaluated by stimulating mouse splenocytes (n = 5 per group) to hBp, vaccine antigens or hBp + antigens, whereas the culture medium was used as a negative control, and 1 μg/mL PWM was used as a positive control. The results showed that the secretion of IFN-γ, IL-17A and IL-5 did not differ significantly among the vaccinated groups except for the media negative control group and PWM control group (*p* > 0.05, Fig. 3A). However, cytokine secretion was significantly different according to the stimulator used; the mean IFN-γ expression levels of the three vaccinated groups, stimulated by hBp + antigen was three times higher than that of the antigen-stimulated group. Furthermore, IL-5 was significantly upregulated in the groups stimulated by the vaccine antigens, while limited responses were observed when stimulated by hBp (Fig. 3A,B). On the other hand, IL-17A had no responses because the negative control group overexpressed by non-specific inflammation response by all three stimulators. In this study, the CMI of the vaccinated groups confirmed Th1/Th2 immunity in both hBp + antigen and antigen stimulator (Fig. 3B). The immunity polarization was calculated with mean cytokine levels of three vaccinated groups (positive control and two study groups) that were baseline corrected with media stimulated results and negative control (saline injected) group results. When hBp stimulator used, only IFN-γ showed response as 3582.48 pg/mL in the three vaccinated groups (Fig. 3B). The mean cytokine response of the three vaccinated groups was 6826.51 pg/mL and 1247.40 pg/mL for IFN-γ and IL-5, respectively when stimulated with hBp + antigen (Fig. 3B). IFN-γ showed 1943.97 pg/mL and IL-5 showed 1773.25 pg/mL when stimulated with antigen (Fig. 3B).

### Bacterial clearance in lung

**Fig. 4:**
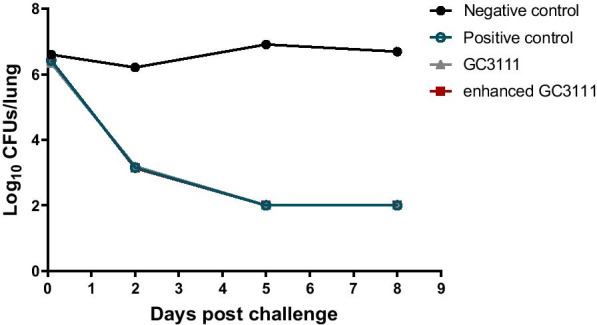
Bacterial clearance in lung. Lungs were extracted from the mice subjected to the challenge test, and the pertussis bacterial colonies were enumerated at 2 h and 2, 5, and 8 days after the challenge. Four mice were sacrificed at each time points. By Day 5 post-challenge, the bacterial colonies were hardly found in any of the groups, with the exception of the negative control group, which was injected with saline solution

The vaccine efficacy was evaluated against the clinical pertussis strain. Four mice was sacrificed at each time points per group. The results from the test using the clinically isolated strain showed that *B. pertussis* was removed quickly in the lungs and was almost eliminated after 5 days (Fig. 4). The results were the same in the two study groups and the positive control group. Compare to 2 h after intranasal challenge, the CFUs of *B. pertussis* decreased at day 2 in the study groups and positive control group (Fig. 4). This result showed protective efficacy against *B. pertussis* in both the positive group and the two study groups while the negative control group retained bacterial CFUs during the test period and showed even more CFUs than 2 h after challenge.

## Discussion

Since 2000, a serological study in Korea has confirmed incidents of pertussis infection with higher certainty than reported earlier, and small-scale pertussis outbreaks have occurred once every 3 years since 2009, leading to the requirement for Tdap vaccination. In 2010, GC Pharma, a national company, started developing Tdap and DTaP vaccines; and our laboratory, the Vaccine Bio Research Institute, conducted animal-based studies [15, 16] and performed clinical trials [14] using the Tdap booster vaccine. In animal studies, GC Pharma’s new Tdap vaccine, GC3111, was compared with Boostrix™, a commercially available product in Korea. The humoral immunity was assessed after a single dose of DTaP vaccine followed by Tdap booster vaccine [15], and CMI was assessed after two doses of DTaP vaccine followed by Tdap booster vaccine [16]. After two animal studies and clinical trials, GC Pharma complemented GC3111 to improve the anti-FHA antibody response. The present study aimed to show the immunogenic response and efficacy of complemented GC3111 (enhanced GC3111) compared to Boostrix™ and the former GC3111 vaccine and to verify anti-FHA response reinforcement.

The protective effects of the humoral response to the aP vaccine were actively investigated soon after aP vaccine development, and the importance of the humoral response to PT, PRN, FHA, and fimbriae antigens was evaluated in different systems, including animal models [18–20]. Humoral immunity to these antigens of *B. pertussis* is known to protect the individual from pertussis infection by neutralizing the pathogenic antigens or by activating the complement system that activates CMI [20–22]. Among the immunogens present in the vaccine, PT is known as the most important immunogen and induces the generation of protective antibodies that provide direct protection from pertussis infection [23, 24]. Moreover, the humoral immunity generated by aP-vaccinated pregnant women can prevent infants from pertussis infection since the antibodies produced by the mother can deliver to the foetus [25–28]. Thus, evaluation of humoral response after pertussis vaccination is immensely significant. In this study, compared to the positive control, the GC3111 group showed lower anti-FHA antibody level 2 weeks after booster vaccination, which was in line with the observations of a previous study [14]; however, the antibody response was comparable between the enhanced GC3111 group and the positive control group with respect to all antigens and all time points (Fig. 2B). Hence, our results verified that the humoral immune response was improved with the enhanced GC3111 Tdap vaccine. With respect to the effect of two doses of DTaP vaccination, anti-PT, anti-FHA, and anti-PRN antibody titres were elevated even before the booster vaccination, and these levels were further enhanced after the booster vaccination and retained at a high level until 4 weeks after vaccination (Fig. 2B). Considering the humoral responses to tetanus and diphtheria, all groups except the negative control group revealed a titre of over 0.1 U/mL (the protective level) from 1 week before booster vaccination, and this is predicted by two doses of DTaP vaccination (Fig. 2A).

The fact that CMI plays crucial roles in preventing pertussis infection was first shown in a mouse model in 1993 [29] followed by clinical experiments [30]. The importance of both Th1 [31, 32] and Th17 [33–35] type CMI responses was confirmed in animal and clinical studies. IFN-γ and IL-17 are the main cytokines that provide crucial protection. Recently, the resurgence of pertussis outbreaks [1–3] and the protective effects of the aP vaccine and whole cell pertussis (wP) vaccine [36] were compared frequently. This was based on the observations of some researchers who postulated that the wP vaccine induces Th1/Th17 responses similar to natural infection [33, 37, 38], whereas the aP vaccine mainly induces the Th2 response, resulting in a weaker protective effect than that of the wP vaccine. Previous studies showed that the aP vaccine generated Th1/Th2 [39] or Th2/Th17 [38, 40] responses. This inconsistency in the findings may be attributed to the difference in animal models used in the studies [41] as well as the study design, such as vaccine schedule and stimulation condition. In general, the Th2 dominant response is the common CMI in aP vaccine-based studies. In this study, hBp was included as one of the simulators to indirectly examine the effects of natural exposure, while vaccine antigens were used as simulators to evaluate the response to the aP vaccine. After exposure to the stimulators, IFN-γ, IL-17, and IL-5 showed significant differences between stimulators that were used but no differences between the positive control group and study groups (Fig. 3A). In the negative control group, non-specific inflammation response was observed in the IFN-γ and IL-17 cytokines. Particularly, IL-17 cytokine showed no responses because the negative control group and vaccinated groups had similar results regardless of the stimulators (Fig. 3A). Notably, in the hBp stimulator group, we observed only IFN-γ responses (Fig. 3B). In the hBp + antigen or antigen stimulator group, the levels of IFN-γ and IL-5 were significantly higher compared to the saline injected negative control group, indicating that Th1 (IFN-γ)/Th2 (IL-5) adaptive immunity (Fig. 3B). This result is consistent with previous studies showing that the aP vaccine primarily induces the Th2 response but induces a dominant Th1 response when exposed to natural pertussis [42–45]. However, in this study, there are some limitations due to the in vitro system, and natural pertussis exposure could be substituted by hBp indirectly.

In addition, the results of the bacterial challenge test using the clinical pertussis strain for real and reliable assessment showed that all vaccinated groups cleared the pathogen from Day 2 post-challenge, and by Day 5, the pathogen was hardly found in the lungs, thereby confirming the similar efficacy of the booster vaccines in the three vaccinated groups (Fig. 4).

## Conclusions

GC Pharma’s enhanced GC311 Tdap vaccine addresses the limitations of the previous GC311 Tdap vaccine, wherein a lower anti-FHA antibody response is observed compared to that of the commercially available product. Our study outcomes confirmed that after booster vaccination, the humoral as well as the CMI responses were comparable to those of the commercially available product with equivalent efficacy against the clinical strain. Our findings present strong evidence that similar findings may be obtained in the phase II clinical trial that is currently being carried out.

## Supplementary Information

**Additional file 1.** Table S1. Raw data of cell mediated cytokine immune response by cytokine ELISA kit.

**Additional file 2**. Table S2. *B. pertussis* clearance was evaluated. The colony forming cells from lungs were counted two times.

## Data Availability

The datasets used and/or analyzed during the current study are available from the corresponding author on reasonable request.

## Abbreviations

aP: Acellular *Bordetella pertussis*
CFU: Colony forming unit
CMI: Cell mediated immunity
DT: Diphtheria toxoid
DTaP: Diphtheria-tetanus-acellular pertussis
FHA: Filamentous hemagglutinin
PRN: Pertactin
PT: Pertussis toxin
PWM: Pokeweed mitogen
SEM: Standard errors of the means
Tdap: Tetanus-reduced dose diphtheria-acellular pertussis
TT: Tetanus toxoid
WP: Whole cell pertussis
